# Temporal and spatially controlled APP transgene expression using Cre-dependent alleles

**DOI:** 10.1242/dmm.049330

**Published:** 2022-05-13

**Authors:** Emily J. Koller, Melissa Comstock, Jonathan C. Bean, Gabriel Escobedo, Kyung-Won Park, Joanna L. Jankowsky

**Affiliations:** 1 Department of Neuroscience, Huffington Center on Aging, Baylor College of Medicine, Houston, TX 77030, USA; 2 Departments of Neurology, Neurosurgery and Molecular and Cellular Biology, Huffington Center on Aging, Baylor College of Medicine, Houston, TX 77030, USA

**Keywords:** Alzheimer's disease, Amyloid β, Amyloid precursor protein, Cre-dependent, Transgenic mouse

## Abstract

Although a large number of mouse models have been made to study Alzheimer's disease, only a handful allow experimental control over the location or timing of the protein being used to drive pathology. Other fields have used the Cre and the tamoxifen-inducible CreER driver lines to achieve precise spatial and temporal control over gene deletion and transgene expression, yet these tools have not been widely used in studies of neurodegeneration. Here, we describe two strategies for harnessing the wide range of Cre and CreER driver lines to control expression of disease-associated amyloid precursor protein (APP) in modeling Alzheimer's amyloid pathology. We show that CreER-based spatial and temporal control over APP expression can be achieved with existing lines by combining a Cre driver with a tetracycline-transactivator (tTA)-dependent APP responder using a Cre-to-tTA converter line. We then describe a new mouse line that places APP expression under direct control of Cre recombinase using an intervening lox-stop-lox cassette. Mating this allele with a CreER driver allows both spatial and temporal control over APP expression, and with it, amyloid onset.

This article has an associated First Person interview with the first author of the paper.

## Abstract (precis)

**Summary:** We describe two strategies and a new mouse allele for improved spatial and temporal control over transgene expression using Cre/CreER drivers in models of Alzheimer's disease and other neurodegenerative disorders.

## INTRODUCTION

Over the past 25 years, nearly 100 transgenic mouse lines have been developed to overexpress the amyloid precursor protein (APP) (AlzForum.org). Most of these models carry one or more familial mutations associated with Alzheimer's disease (AD) and are designed to recapitulate the cerebral amyloid β (Aβ) amyloidosis that characterizes this dementia. Because the degree of APP overexpression – along with the mutations expressed – govern the amount and form of Aβ produced and therefore dictate the rate of amyloid deposition, many of these models use transgene promoters chosen for their ability to produce stable and high expression levels throughout the adult brain (Jankowsky and Zheng, 2017). Common transgene promoters include those from the prion protein (PrP), Thy1 cell surface antigen (Thy1) and platelet derived growth factor subunit B (PDGFβ), each active in multiple cell types in the central nervous system (CNS) and beyond (Sasaguri et al., 2017). Newer knock-in models use the endogenous APP promoter to achieve spatiotemporal precision in the expression of the APP protein, but must overcome the endogenous limit on Aβ production by incorporating multiple familial mutations and homozygosing the targeted allele to generate amyloid pathology in an experimentally tractable timeframe (Saito et al., 2014).

Two limitations arise with the use of standard transgene promoters such as PrP, PDGFβ or Thy1. The first limitation is that there is no control over which cell types express the transgene. The promoter used in the transgene construct dictates which cells may express the protein of interest, but as clearly demonstrated by the series of Thy1-YFP lines generated by Feng and colleagues, transgene integration site can further restrict this potential (Feng et al., 2000). Current amyloid models offer no way to direct transgenic APP to specific cell types for studies that might need this specificity, such as a comparison of APP processing in excitatory versus inhibitory neurons, or assessment of amyloid formation by layer 2/3 versus layer 5 pyramidal neurons. Researchers working in other fields have made strong use of the Cre-loxP system to achieve such cell-type specificity, but the Alzheimer's field has no equivalent Cre-dependent APP transgenic lines (but see Baglietto-Vargas et al., 2021; Chabrier et al., 2014). While Cre lines are most commonly associated with gene deletion, an appropriately designed responder line can use recombination to drive gene onset by excision of a loxP-flanked stop cassette (i.e. Allen Institute Cre-reporter lines; Madisen et al., 2010). The Jackson Laboratory alone offers over 300 Cre driver lines for gene expression in a wide variety of cell types, but this resource cannot be tapped for Aβ research without appropriate loxP-containing responder lines.

The second limitation of standard transgenic models is the lack of temporal control over gene expression. The most common promoters for APP models turn on embryonically and remain active throughout life (Jankowsky and Zheng, 2017). Amyloid formation in humans usually begins after 50 years of age, yet our most aggressive mouse models develop plaques before or soon after they can breed (Chishti et al., 2001; Huichalaf et al., 2019; Oakley et al., 2006; Rodgers et al., 2012). Having the means to delay transgene onset might improve our modeling of this disease. Temporal control was introduced into Cre-dependent expression systems with the advent of CreER, which combined Cre with a modified estrogen receptor (Feil et al., 1997). Recombinase activity in CreER animals is activated by exogenous administration of the synthetic estrogen receptor modulator tamoxifen, which allows the timing of gene excision or expression to be extrinsically controlled (Feil et al., 2009). However, lacking a Cre-dependent APP responder line, the Alzheimer's field has not yet been able to utilize the wide range of CreER drivers available for combined spatial and temporal control over transgene expression. Alzheimer's researchers have instead used the tetracycline-transactivator (tTA) system to control the timing of transgene onset (Jankowsky et al., 2005; Liu et al., 2015; Maeda et al., 2016; SantaCruz et al., 2005). Unlike CreER, the tTA system allows transgene expression to be both activated and inactivated using the drug doxycycline (Bejar et al., 2002; Rodgers et al., 2012). A handful of tTA driver lines exist for use in the CNS, but the list of available tTA drivers is much smaller than for Cre, making this system somewhat limited for regional or cell-type-specific studies (Hoover et al., 2010; Lin et al., 2009; Mayford et al., 1996; Song et al., 2012; Walker et al., 2015; Wang et al., 2008; Yasuda and Mayford, 2006).

Both the spatial and temporal limitations of standard APP transgenic models could be overcome by the creation of a Cre-dependent APP allele. An alternative strategy that takes advantage of existing transgenic lines would use a Cre-to-tTA converter allele to yield Cre-dependent expression of a tetracycline (tet)-regulated transgene (Li et al., 2010; Wang et al., 2008). Each approach would allow users to tap into the vast array of Cre driver lines for precise cellular control over transgene expression. Additional temporal control over transgene onset could be achieved using doxycycline, or more simply using CreER drivers in place of Cre. Here we tested each of these approaches for temporal and spatial control over transgenic APP expression to model the brain amyloidosis of Alzheimer's disease. Our findings highlight the challenges in developing new models with added temporal or cellular specificity and yield recommendations for future work. Most importantly, we describe a new Cre-dependent APP allele that allows researchers to finally harness the vast repository of Cre and CreER lines available for neuroscience.

## RESULTS

### Combining mouse lines to achieve Cre-dependent doxycycline-controlled APP expression

**Fig. 1.**
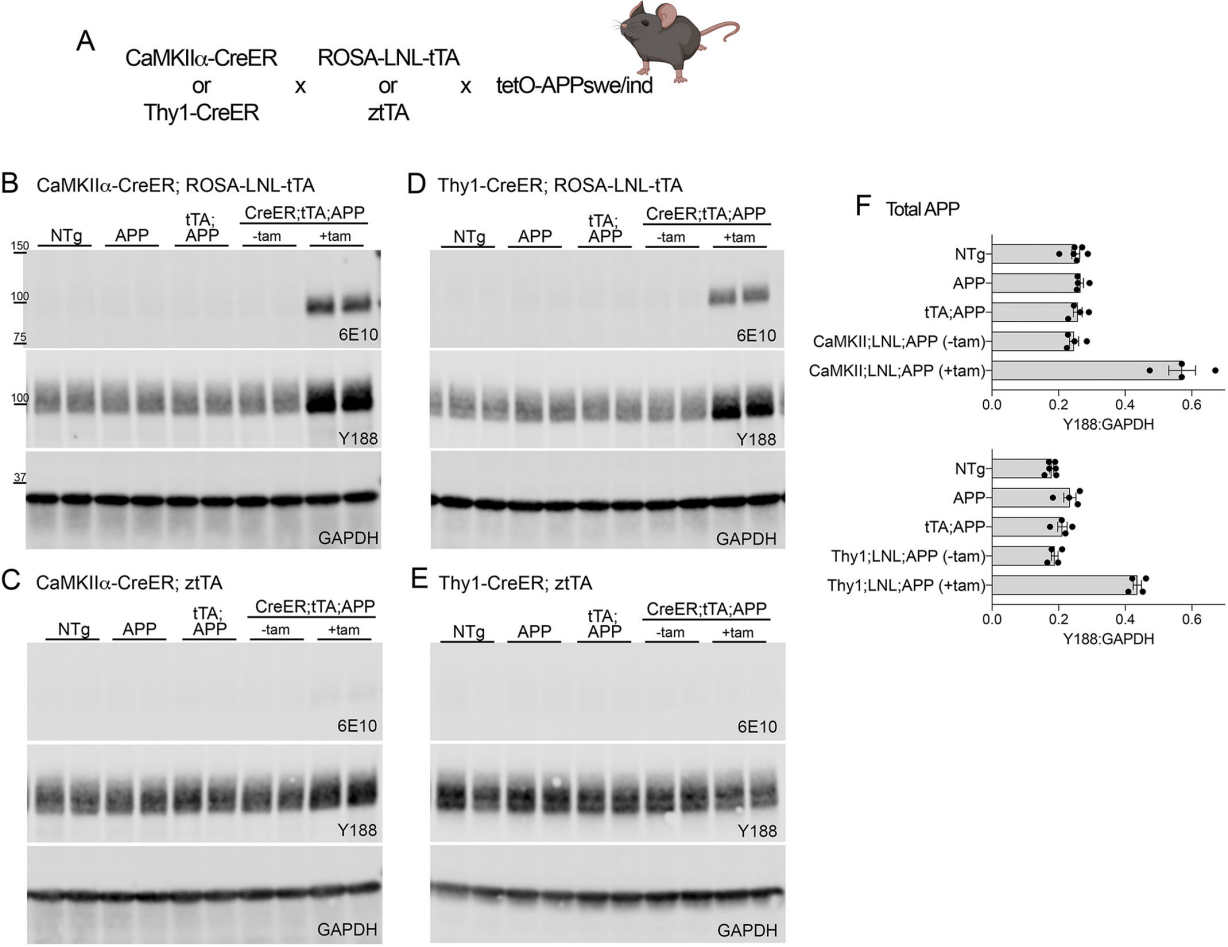
**Characterization of triple transgenic models for Cre-dependent APP expression based on existing mouse lines.** (A) Diagram of CreER×LSL-tTA×tetO-APP breeding strategy indicating CreER driver lines and Cre-to-tTA converter lines that were tested. (B-E) Western blots for human APP (6E10), total APP (Y188) and internal control GAPDH on cortical homogenates from each of the four transgenic combinations tested: CaMKIIα-CreER;ROSA-LNL-tTA;tetO-APP (B), CaMKIIα-CreER;ztTA;tetO-APP (C), Thy1-CreER;ROSA-LNL-tTA;tetO-APP (D), Thy1-CreER;ztTA;tetO-APP (E). Each blot includes four genotypes: non-transgenic (NTg), tetO-APP single transgenic (Tg) (APP), LSL-tTA+tetO-APP double Tg (tTA;APP) and the triple transgenic CreER+LSL-tTA+tetO-APP (CreER;tTA;APP). Triple transgenic mice were harvested at 10 weeks of age with and without tamoxifen treatment 2 weeks earlier (labeled as ±tam). (F) Quantitation of total APP relative to GAPDH for the two ROSA-LNL-tTA triple transgenic models. Upper graph: CaMKIIα-CreER;ROSA-LNL-tTA;tetO-APP; lower graph: Thy1-CreER;ROSA-LNL-tTA;tetO-APP. *n*=4 mice for all groups except NTg (*n*=6). Graphs show mean±s.e.m.

Our goal in these studies was to create a model in which we could harness Cre driver lines for improved spatial and temporal control over transgenic APP for more precise studies on the impact of cellular origin and timing of pathogenic APP exposure. Our initial characterization of this approach focused on Cre drivers with widespread neuronal expression patterns so that we could use simple outcome measures such as cortical transgene expression level or amyloid onset to determine the efficiency of our crosses. We began our studies by taking advantage of existing mouse lines that could be combined to achieve Cre-dependent expression of a tTA-controlled APP allele. The work was framed around tetO-APP line 102 expressing the Swedish and Indiana mutations (Jankowsky et al., 2005). In past work, this tetO-APP line had been bred directly with the very strong CaMKIIα-tTA driver line to elicit amyloid plaques within 1-2 months of birth (Huichalaf et al., 2019; Rodgers et al., 2012). Here we wanted to control APP expression with Cre and thus relied on a Cre-to-tTA converter line to translate Cre activity into tTA expression. We tested two converter lines designed with a loxP-flanked stop cassette (loxP-stop-loxP, LSL; or loxP-neomycin-loxP, LNL) in front of the tTA coding sequence so that expression of tTA was only induced in cells in which Cre was active (Li et al., 2010; Wang et al., 2008). Both converter lines were targeted to the ROSA26 locus (R26 or ROSA). The ztTA converter line included the CAG promoter before the LSL cassette, whereas the ROSA-LNL-tTA relied solely on the endogenous ROSA26 promoter for expression. Both the CAG promoter, which is a synthetic element composed of the chicken β-actin promoter and the CMV enhancer, and the ROSA26 endogenous promoter can be widely expressed throughout the body. These promoters therefore are appropriate for use with a wide variety of Cre driver lines. These converter lines were each in turn tested with two Cre driver lines, one controlled by the CaMKIIα promoter, the other by the Thy1.2 promoter (Erdmann et al., 2007; Young et al., 2008). Both driver lines are active primarily (Thy1.2) or exclusively (CaMKIIα) in neurons, and expressed the inducible CreER^T2^ recombinase that allowed us to control the onset of Cre activity by systemic administration of tamoxifen (tam). Interbreeding of these two CreER drivers with the two Cre-to-tTA converters generated four transgene combinations that were tested with the tetO-APP responder line (Fig. 1A). All alleles were used in the heterozygous state and on a mixed B6;ICR genetic background. The use of an outbred ICR/CD1 background was required for survival of the ztTA line (D. C.-H. Wang, Stanford University, personal communication), but eased breeding for all combinations tested.

We first tested whether the inducible Thy1-CreER and CaMKIIα-CreER lines produced the expected neuronal expression patterns upon tam exposure using fluorescent Cre reporter lines. We also examined the degree of tam-independent leaky expression allowed by each driver. The eYFP reporter line Ai3 was used with the CaMKIIα-CreER, and the tdTomato reporter line Ai14 was used with the Thy1-CreER as this driver carries eYFP alongside CreER. We found that both CreER lines produced sparse cortical expression in the absence of tam and both showed strong fluorescence throughout the forebrain upon tam treatment (Figs S1 and S2). Three main factors distinguished expression patterns in the two drivers. First, there was significant tam-independent fluorescence in the hippocampus of the CaMKIIα-CreER line that was largely absent from the Thy1-CreER line. Hippocampal fluorescence increased substantially upon tam administration in both lines, but the baseline amount of tam-independent expression was higher with CaMKIIα-CreER. Second, the CaMKIIα-CreER expressed more strongly in striatal neurons than did Thy1-CreER. Third, Thy1-CreER was active in the cerebellum and hindbrain, whereas CaMKIIα-CreER was not. These findings indicated that both lines would be suitable for driving APP expression in models of CNS amyloidosis, but that hippocampal leaky expression should be examined when using the CaMKIIα-CreER driver.

We next tested the four combinations of CreER, tTA and APP alleles by western blot for the levels of transgenic APP attained following tam treatment and for the levels of expression observed in the absence of tam. Leaky expression in the absence of tam would affect the precision of temporal control, whereas the expression levels attained after tam exposure would affect the rate at which amyloid deposits appeared after transgene onset. Total APP was detected with the antibody Y188 and human transgene-specific APP was detected with the 6E10 antibody. We saw no transgenic APP expression in cortical extracts from any of the control conditions, up to and including triple transgenic mice without tam (Fig. 1B-E). In the absence of tam, the hippocampus was also free of transgenic APP, even in mice controlled by the CaMKIIα-CreER driver, which showed tam-independent leakiness with the fluorescent reporter (Fig. S3A,B). Following tam administration, we observed clear APP induction in two of the four CreER;tTA;APP combinations tested. Both Thy1- and CaMKIIα-CreER drivers induced transgenic APP expression when used with the ROSA-LNL-tTA converter line (Fig. 1B,D; Fig. S3A,B). Cortical transgene levels were 2.3-fold higher than endogenous APP in the CaMKIIα-CreER;ROSA-LNL-tTA;tetO-APP model and 2.4-fold higher than endogenous APP in the Thy1-CreER;ROSA-LNL-tTA;tetO-APP mice (Fig. 1F). Hippocampal transgene levels were similar at 2.9-fold higher than endogenous APP for CaMKIIα and 2.3-fold higher for Thy1 (Fig. S3C,D). The other two CreER;tTA;APP transgene combinations based around the ztTA converter produced no transgenic APP expression whatsoever following identical tam treatment (Fig. 1C,E). This outcome was unexpected as past work had shown the ztTA converter line to be more efficient for tTA-dependent gene expression than the ROSA-LNL-tTA converter, yet here it was completely ineffective (Madisen et al., 2015).

### A new APP transgenic model for direct Cre control

Past studies have shown that expression levels roughly 5- to 10-fold over endogenous levels are needed for APP transgenes to produce amyloid in less than a year, depending on the number and identity of mutations included in the transgene (Chishti et al., 2001; Games et al., 1995; Hsiao et al., 1996). Although our use of converter lines to translate Cre activity into tTA signal worked in principle, the levels of transgenic APP expression attained at 2- to 3-fold over endogenous levels were too low to produce amyloid deposits as rapidly as we had hoped. The relatively mild transgene levels could be an advantage for studies on protein seeding in which a model lacking innate pathology is desired, or for studies on circuit function in which changes in circuit formation due to APP overexpression would confound outcome measures. However, this expression level would be too low for many other studies in which amyloid pathology is desired. We therefore set out to create a Cre-dependent APP transgenic allele that could be directly activated by Cre or CreER without the need for a converter line.

**Fig. 2.**
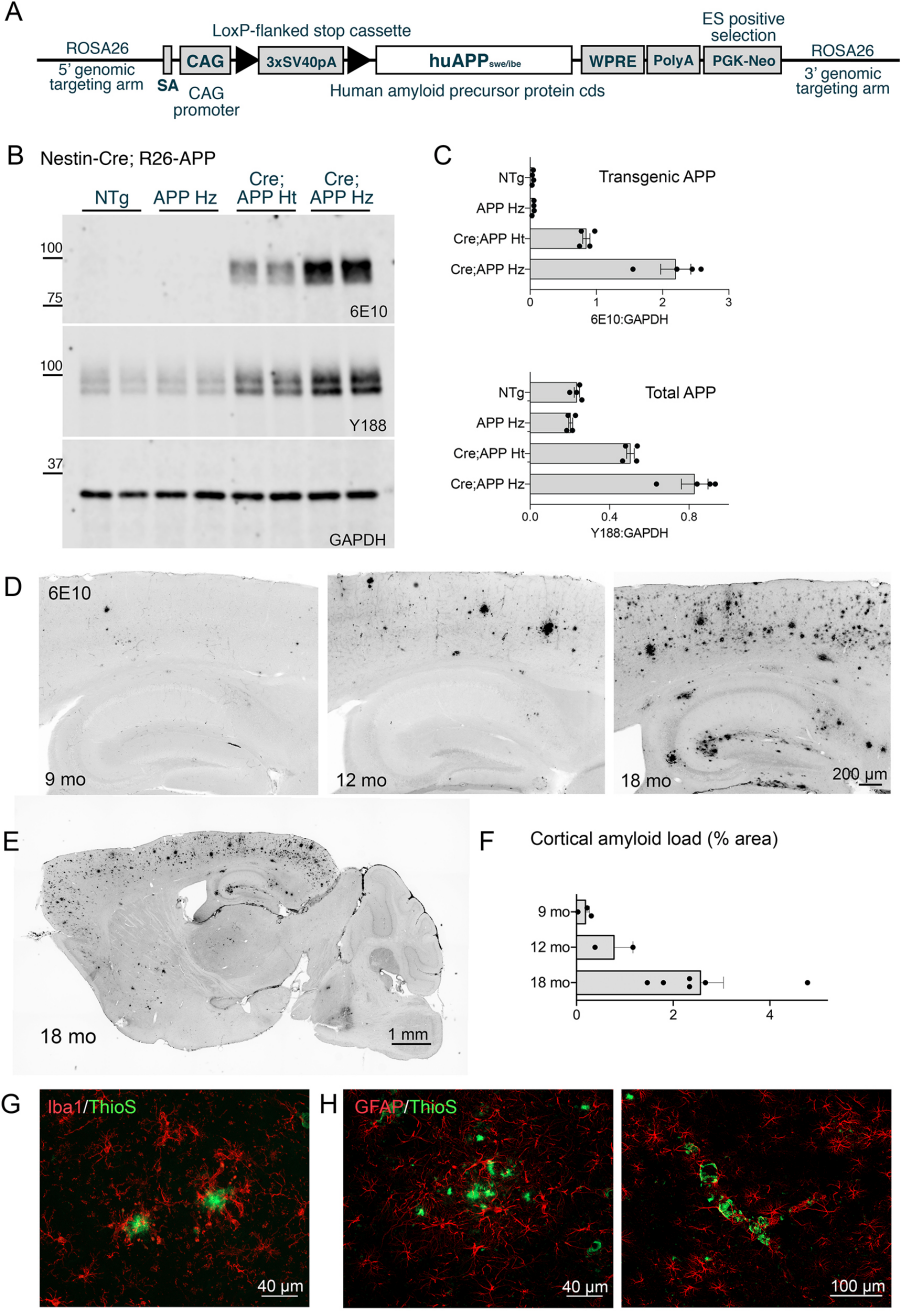
**Construction and characterization of a new Cre-dependent APP transgenic mouse line.** (A) Diagram of main sequence elements in the ROSA26-CAG-LSL-APP^Swe/Ibe^ targeting construct. The transgene was targeted to the ROSA26 locus where the splice acceptor sequence (SA) allowed transcription from the endogenous promoter. Expression was enhanced by addition of a CAG promoter prior to the loxP-flanked stop cassette, which was followed by the human APP 695 coding sequence encoding Swedish and Iberian mutations. The woodchuck hepatitis virus post-transcriptional regulatory element (WPRE) was used to stabilize transcribed mRNA, followed by the bovine growth hormone poly A signal and neomycin selection marker. (B) Western blots for human APP (6E10), total APP (Y188) and internal control GAPDH on cortical homogenates from Nestin-Cre×R26-APP offspring. Four genotypes of mice were tested: NTg, R26-APP homozygous mice without Cre (APP Hz), Nestin-Cre;R26-APP heterozygous mice (Cre;APP Ht) and Nestin-Cre;R26-APP homozygous mice (Cre;APP Hz). (C) Quantitation of transgenic (6E10, upper panel) and total APP (Y188, lower panel) relative to GAPDH for each genotype. (D) 6E10 immunostaining for amyloid deposits in Nestin-Cre;APP-LSL-APP homozygous mice at 9, 12 and 18 months (mo) of age. Scale bar: 200 µm. (E) Tiled image of 6E10 immunostaining from an 18 month-old Nestin-Cre;APP-LSL-APP homozygous mouse illustrates the distribution of plaques throughout the brain. Scale bar: 1 mm. (F) 6E10-stained sections were used to measure cortical amyloid load at 9, 12 and 18 months as a fraction of the total surface area. (G) Co-immunostaining for Iba1 (red) with Thioflavin S (ThioS, green) reveals the presence of reactive microglia surrounding fibrillar plaques at 18 months of age. Scale bar: 40 µm. (H) Reactive astrocytes stained with GFAP (red) were also found around fibrillar plaques (ThioS, green). Astrocytosis was found both in the vicinity of amyloid plaques (left) and along areas of vascular amyloid (right). Both images were taken from 18 month-old animals. Scale bars: 40 µm (left), 100 µm (right). For western blotting, *n*=4 mice for all groups. For amyloid histology, *n*=3 mice at 9 months, *n*=2 mice at 12 months, *n*=6 mice at 18 months. Graphs show mean±s.e.m.

We built our Cre-dependent human APP transgene based on constructs developed by the Allen Institute for their Cre-dependent eYFP reporter line Ai3. Our construct placed human APP 695 encoding the Swedish and Iberian mutations under control of the CAG promoter (Fig. 2A). An intervening loxP-flanked stop cassette prevented transgene expression in the absence of Cre. The entire construct was targeted to the ROSA26 locus with genomic homology arms. The plasmid was electroporated into C57BL/6N embryonic stem cells and correctly targeted clones produced a founder male that was bred to generate a R26-APP^Swe/Ibe^ colony.

Offspring of this founder animal were mated with a Nestin-Cre driver line to measure transgenic APP expression in the brain. Nestin is expressed in all CNS neural precursors during maturation and was used here to drive widespread neuronal Cre expression (Tronche et al., 1999; Zimmerman et al., 1994). Although we saw no expression in the absence of Cre, the level of transgenic APP in mice heterozygous for the R26-APP allele was just 2.2-fold over endogenous levels, roughly the same as we had achieved with the converter line and the tetO-APP transgene (Fig. 2B,C). Consistent with this low level of APP overexpression, we found no signs of amyloid pathology in Nestin-Cre;R26-APP heterozygous mice at 6 or 12 months of age. We therefore homozygosed the R26-APP allele in Nestin-Cre mice, and increased cortical transgene expression from 2.2- to 3.5-fold over endogenous levels (Fig. 2B,C). This was sufficient to produce initial amyloid deposits by 9 months of age and widespread pathology by 18 months (Fig. 2D-F). Plaques first appeared in the cortex and later in the hippocampus, with mild pathology in the striatum and very limited deposits in the thalamus, cerebellum and brain stem by 18 months of age. Thioflavin S-positive deposits were present from the earliest signs of amyloid formation at 9 months and increased in size and number with age. Thioflavin S staining also revealed the presence of cerebral amyloid angiopathy by 9 months and was found primarily in leptomeningeal arteries and penetrating cortical arterioles. This model also displayed pronounced microgliosis and astrocytosis. Microgliosis was seen around Thioflavin S-positive plaques by 9 months of age. The intensity of Iba1 staining unexpectedly waned by 18 months, such that most plaques had a few Iba1-stained microglia nearby, but not the clear accretion of cells observed upon amyloid growth in other APP transgenic models (Hickman et al., 2008; Matsuoka et al., 2001; Wang et al., 2011) (Fig. 2G). Reactive astrocytes were also found surrounding fibrillar plaques and were prominent around the vascular amyloid (Fig. 2H) such that glial fibrillary acidic protein (GFAP) immunostaining could be used as a surrogate for cerebral amyloid angiopathy (CAA) at all ages examined.

### Using CreER to achieve temporal control over APP expression with the new allele

**Fig. 3.**
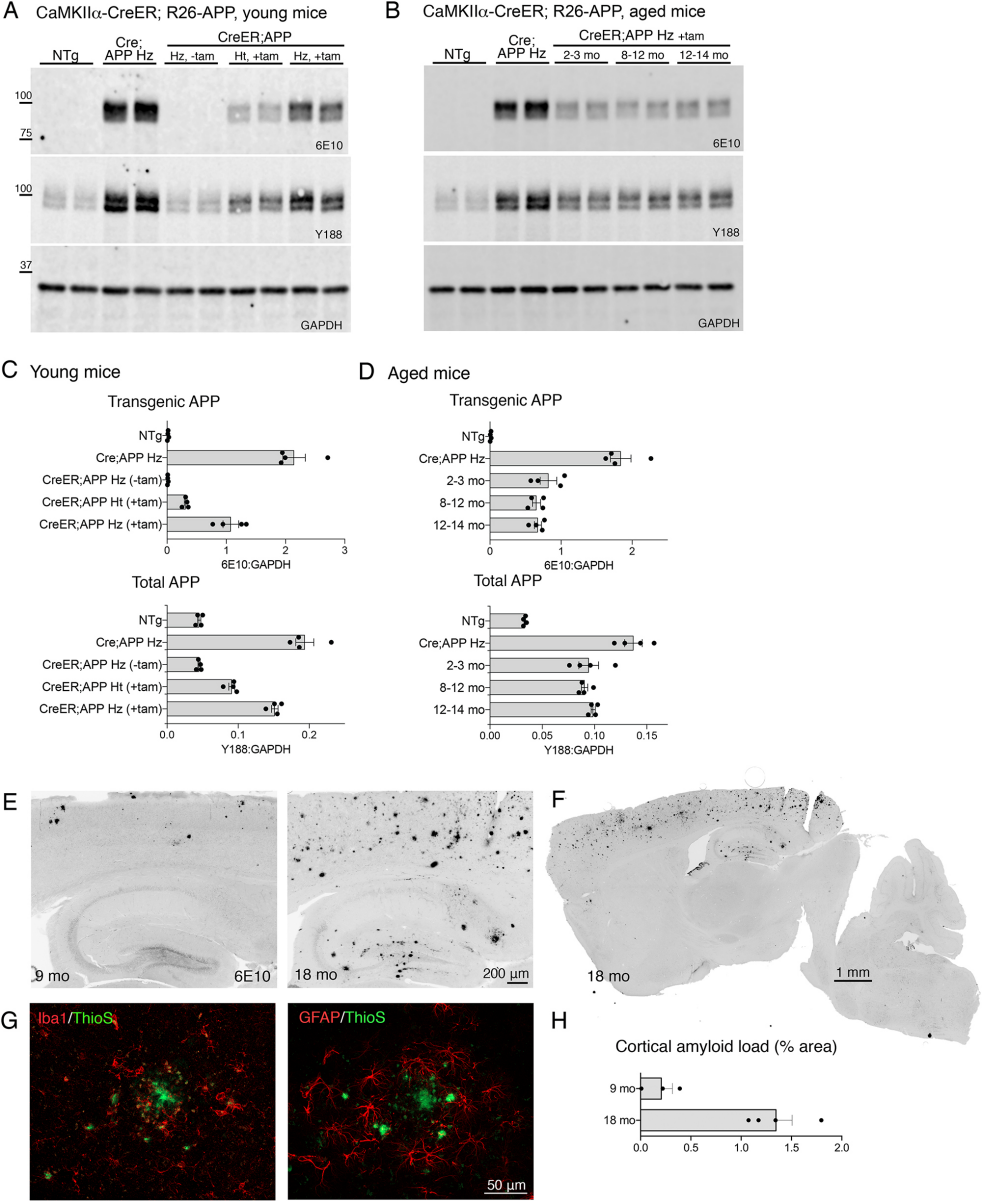
**Tamoxifen-inducible CreER expression of the new R26-APP allele is less efficient than Cre itself, but does not change with age.** (A,B) Western blots for human APP (6E10), total APP (Y188) and internal control GAPDH on cortical homogenates from R26-APP mice co-expressing Nestin-Cre or CaMKIIα-CreER. NTg and Nestin-Cre;R26-APP homozygote animals (Cre;APP Hz) served as controls. CaMKIIα-CreER;R26-APP mice were tested with tam (+tam) and without tam (−tam), either as heterozygotes for APP (Ht) or homozygotes (Hz) (A), or with tam treatment initiated at different ages (B). (C,D) Quantitation of transgenic (6E10, upper panel) and total APP (Y188, lower panel) relative to GAPDH for each genotype and treatment condition from panel A (C) or panel B (D). (E) 6E10 immunostaining for amyloid deposits in CaMKIIα-CreER;R26-APP homozygous mice harvested at 9 and 18 months after tam injection at 8 weeks of age. Scale bar: 200 µm. (F) Tiled image of 6E10 immunostaining from a CaMKIIα-CreER;R26-APP homozygous mouse harvested 18 months after tam administration illustrates the distribution of plaques throughout the brain. Scale bar: 1 mm. (G) Co-immunostaining for Iba1 (red, left panel) or GFAP (red, right panel) with Thioflavin S (green) reveals gliosis around fibrillar plaques at 18 months of age. Scale bars: 50 µm. (H) 6E10-stained sections were used to measure cortical amyloid load following 9 and 18 months of transgene expression as a fraction of total surface area. For western blotting, *n*=4 mice for all groups. For amyloid histology, *n*=3 mice for 9 months, *n*=4 mice for 18 months. Graphs show mean±s.e.m.

We next wanted to test whether the new Cre-dependent APP allele could be used with CreER driver lines to allow temporal control over transgene onset in addition to spatial control afforded by the Cre promoter. We therefore mated the R26-APP line with CaMKIIα-CreER mice to test how this combination compared to expression levels in mice driven by Nestin-Cre. This was not an entirely fair comparison as Nestin-Cre expresses in all neurons, whereas CaMKIIα-CreER expresses in a subset; however, both are widely active in forebrain. Consistent with this difference in promoters, Nestin-Cre;R26-APP homozygous animals expressed slightly more transgenic protein than tam-treated CaMKIIα-CreER;R26-APP homozygous mice. Cortical APP expression in homozygous Nestin-Cre;R26-APP mice was 4.3-fold over endogenous levels in this cohort; expression in tam-treated CaMKIIα-CreER;R26-APP homozygotes was 3.4-fold over endogenous levels (Fig. 3A,C). Cortical APP in tam-treated CaMKIIα-CreER;R26-APP heterozygotes was just 2-fold higher than endogenous levels.

We next tested whether the model might be useful for aging studies in which transgene expression was withheld until the mice reached mid-life or later. We therefore administered tam to CaMKIIα-CreER;R26-APP homozygous mice at ages ranging from 8 to 14 months. Transgene expression following tam exposure did not change with age. In this cohort, transgene levels were 2.9-fold over endogenous levels at 2-3 months, 2.8-fold at 8-12 months and 3.0-fold at 12-14 months, compared to 4.1-fold in Nestin-Cre;R26-APP homozygotes used as a control (Fig. 3B,D). One key finding is that age increased the mortality of tam treatment, with 10% of mice dying within 2 weeks when treated at 2-3 months of age, but 50% of mice dying with treatment at 12-14 months.

Finally, we examined how long it would take for tam-induced transgenic APP expression to elicit amyloid deposits in CaMKIIα-CreER;R26-APP homozygous mice. We expected that plaque onset would be earlier in the Nestin-Cre;R26-APP mice than in the CaMKIIα-CreER;R26-APP mice as the nestin promoter is active in a broader population of neurons than CaMKIIα and would therefore be producing Aβ from a greater number of cells. The nestin promoter also begins expression much earlier in life than the age at which we chose to activate CreER in the CaMKIIα model. Despite these differences, plaque onset occurred at roughly the same rate in both the CaMKIIα-CreER;R26-APP and Nestin-Cre;R26-APP homozygous mice. Initial deposits in both models appeared 9 months after transgene onset (Fig. 3E,F,H, compared with Fig. 2D-F); however, plaque load rose more slowly in the CaMKIIα-CreER model. Plaque load in CaMKIIα-CreER;R26-APP homozygote mice was half the amount found in Nestin-Cre;R26-APP homozygotes following 18 months of transgene expression (1.3% of the cortical area in CaMKIIα-CreER;R26-APP versus 2.6% in Nestin-Cre;R26-APP mice). Staining for Thioflavin S revealed a small number of fibrillar plaques across the cortex by 9 months of age, without any vascular amyloid. CAA was present by 18 months, along with an increasing number of fibrillar plaques throughout the cortex and hippocampus. Both astrogliosis and microgliosis were present alongside the Thioflavin S-positive deposits. Iba1 immunostaining detected clusters of activated microglia surrounding Thioflavin S-positive plaques at 9 months that became surprisingly less prominent at 18 months. While clustered cells could still be found at 18 months, Iba1 staining was marked by the appearance of numerous puncta throughout the cortex, which were often co-labeled for Thioflavin S (Fig. 3G). GFAP immunostaining revealed a strong but splotchy astrocytic reaction throughout the cortex that was not focused specifically around Thioflavin S-positive plaques (Fig. 3G). Reactive astrocytes were also seen around penetrating vessels that were again not necessarily co-labeled with Thioflavin S; however, the large, loose clusters of cortical astrocytes were the most prominent aspect of gliosis in this model, and contrasted with a more pronounced response to cerebral amyloid angiopathy in the Nestin-Cre;R26-APP animals.

### APP expression in the new models is comparable to existing amyloid strains

**Fig. 4.**
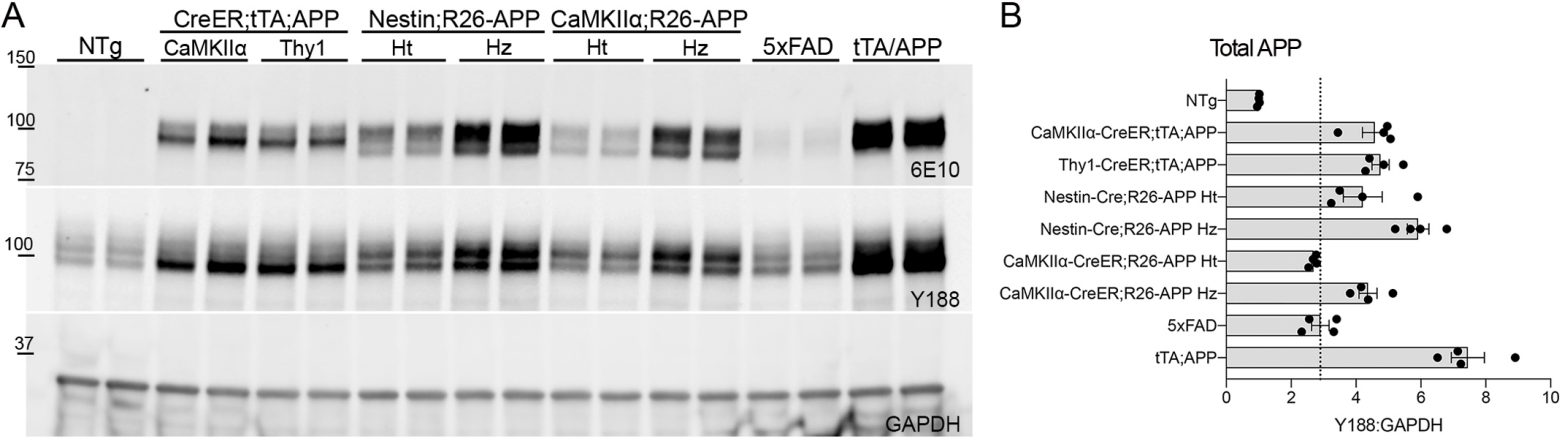
**New constitutive and inducible APP lines exhibit transgene expression comparable to existing APP mouse lines.** (A) Protein expression in cortical homogenates was measured by western blotting for human APP (6E10), total APP (Y188) and GAPDH across eight different APP transgenic mouse lines. Left to right: NTg, CaMKIIα-CreER;ROSA-LNL-tTA;tetO-APP (CAMKIIα), Thy1-CreER;ROSA-LNL-tTA;tetO-APP (Thy1), Nestin-Cre;R26-APP heterozygotes (Ht), Nestin-Cre;R26-APP homozygotes (Hz), CaMKIIα-CreER;R26-APP heterozygotes, CaMKIIα-CreER;R26-APP homozygotes, 5×FAD and CaMKIIα-tTA;tetO-APP (tTA/APP) mice. (B) Quantitation of total APP relative to GAPDH, normalized to NTg. The dotted line indicates the average expression level of total APP in 5×FAD mice. *n*=4 mice for all groups. Graph shows mean±s.e.m.

To better place our new Cre-dependent models into the context of existing APP transgenic strains, we performed one final western blot to measure cortical APP expression in each of our new lines against the 5×FAD and tTA/APP strains (Jankowsky et al., 2005; Oakley et al., 2006). We chose these two lines for comparison because they use similar transgenic promoters as our new models: 5×FAD expresses human APP and presenilin-1 (PS1) under control of the Thy1 promoter, whereas the tTA/APP strain is under control of the CaMKIIα promoter. Relative APP expression in each of the new lines was consistent with our earlier results. The highest expressing model was the Nestin-Cre;R26-APP homozygote, followed by the CaMKIIα-CreER;R26-APP homozygote. To our surprise, we found that all of our models expressed as much or more transgenic APP as the 5×FAD line, and in several cases expressed nearly as much as the tTA/APP strain (Fig. 4A,B). Our findings suggest that the relative speed of amyloid formation in the 5×FAD line is driven not by the degree of APP overexpression relative to wild-type mice at just 2.9-fold over endogenous levels, but by the co-expression of a dual mutant presenilin with APP encoding the Swedish, Florida and London mutations. This allelic combination increases the ratio of Aβ42:40 to accelerate aggregation. In contrast, the tTA/APP model carries the single APP transgene with Swedish and Indiana mutations, but its expression is enhanced by amplification inherent in the tet-transactivator system. In examining this blot, we were surprised at the relatively low level of transgenic APP detected in the 5×FAD animals as the original description of this line shows much stronger APP overexpression, albeit using a different antibody for APP detection. To determine whether our colony had somehow lost transgene copies with generations of breeding, we sourced 5×FAD mice from an independent colony and indeed confirmed that cortical APP expression is just two to three times higher than endogenous levels (Fig. S4). We conclude that our new Cre-inducible models express transgenic APP at levels comparable to other amyloid mouse strains and that amyloid onset in each model is dictated by the combination of expression levels, the presence or absence of mutant presenilin and the specific APP mutations encoded.

## DISCUSSION

We endeavored to bring the vast resource of Cre driver lines into Alzheimer's research for use in manipulating expression of transgenic proteins associated with disease. Our studies tested two strategies for achieving Cre-dependent expression of human APP, one based entirely on existing transgenic lines, and one which required creation of a new responder line. We show that both approaches achieved Cre-dependent APP expression, and with that achievement, open the promise of harnessing myriad Cre drivers for cell-type and spatial specificity. We further show that CreER drivers can be used for temporal control over transgene onset, opening another untapped resource for Alzheimer's research. The models based on combining Cre with tTA provide the added potential to control both transgene onset and suppression with doxycycline. Importantly, this approach for combining Cre drivers with tTA-responsive transgenes can be readily extended to other disease models for which tTA-dependent alleles already exist, including tau, TDP-43, LRRK, SOD1, huntingtin and α-synuclein (Gu et al., 2010; Hoover et al., 2010; Lin et al., 2009; Liu et al., 2015; SantaCruz et al., 2005; Walker et al., 2015; Wang et al., 2008; Xiong et al., 2017; Yamamoto et al., 2000). Of course, we are not the first to use this approach for combining Cre drivers with tTA-responsive transgenes – both of the converter lines were made by groups seeking this flexibility – but it has rarely been used in neurodegeneration research (Wang et al., 2008).

Our studies taught us several important lessons for future work on Cre-dependent proteinopathy models where high expression levels are critical for recapitulating pathology. The first lesson we learned is that all new transgene combinations must be tested empirically. The best laid plans on paper do not always work in practice. This insight came from the comparison of our two converter lines, ztTA and ROSA-LNL-tTA. Past work by Zeng and colleagues tested both converter lines with the same Cre driver and a fluorescence reporter. In their hands, both converter lines worked well, but ztTA was approximately 40% more efficient (Madisen et al., 2015). We therefore expected our ztTA animals to express more highly than those with ROSA-LNL-tTA. Instead, we found that ztTA yielded no expression at all under our conditions. We cannot explain this outcome, but the lack of expression was consistent across both CreER driver lines. This finding emphasizes the importance of testing new transgene combinations empirically rather than relying on expectations.

The second lesson we learned is that Cre reporter lines are valuable for informing where a driver line can express, but not necessarily where it will elicit recombination when crossed with a different responder. The outcome of any Cre-dependent cross is dependent on an interaction governed by both the driver and the responder transgenes. We were surprised at the degree of tam-independent eYFP expression in our CaMKIIα-CreER;Ai3 bigenic animals and expected this would presage leaky expression of transgenic APP when this driver was mated with our tetO-APP line. Instead, we found no evidence of transgenic APP expression in the hippocampus of untreated animals in which the eYFP leaky expression had been so pronounced. In our case, a better test of potential leakiness might have examined each CreER/converter combination with a tTA-dependent reporter instead of a Cre-dependent one. Nevertheless, this finding illustrates how different responder alleles can produce distinct expression patterns when crossed to the same driver line. The driver line will dictate the potential expression pattern, but features of the responder line such as transgene integration site may further limit the final pattern.

The final lesson we learned is that the CAG promoter has the advantage of providing persistent, widespread transgene expression *in vivo*, but does not match the expression levels of traditional transgenesis when used as a single copy targeted to the ROSA26 locus. In our hands the CAG promoter and a single-copy APP insertion attained expression levels just 2-fold over endogenous levels. We increased this to 3.5- to 4-fold by homozygosing the allele, but this level was still far below what has been achieved with traditional transgenesis to produce models such as Tg2576, APP/PS1 or 5×FAD (Hsiao et al., 1996; Jankowsky et al., 2004; Oakley et al., 2006). At 4-fold overexpression with two familial AD mutations, homozygous animals developed amyloid deposits between 9 and 12 months of age. This slow onset may be an advantage for experiments on aggregate seeding or for behavior and electrophysiological studies in which high levels of APP overexpression can be a confounding variable. Future work might follow the lead of Saito et al. (2014) and Xia et al. (2021 preprint) in creating their APP knock-in models, in which they combined three AD mutations – including the Arctic mutation inside the Aβ domain – to dramatically accelerate amyloid formation. Here we chose mutations that reside outside of the Aβ domain with the goal of producing wild-type human Aβ for our studies, and hope that this decision will be an advantage for other investigators.

Although our studies focused on models for controlling expression of pathogenic APP, we anticipate that the approaches taken and lessons learned will be applicable to many other proteinopathies. Mouse lines with tet-promoters are available from The Jackson Laboratory for tau, α-synuclein, LRRK2 and TDP-43, and privately for SOD1, huntingtin and likely others. There are a relatively small number of tTA driver lines available to manipulate these responders, but the approach taken here for harnessing Cre drivers via Cre-to-tTA converters increases the ways in which these tet-responder lines may be used. Use of Cre drivers could facilitate experiments testing the impact of pathogenic proteins in different layers of the cortex, different neuronal subtypes, or even glial versus neuronal expression. The ability to tap into this resource opens a range of experimental questions that can now be accomplished using this strategy, all with existing transgenic lines.

## MATERIALS AND METHODS

### Cloning of R26-LSL-hAPP^Swe/Ibe^ transgene

Human APP^Swe/Ibe^ 695 amino acid cDNA was amplified from the pBR322-APP^Swe/Ind^ plasmid used to generate the Tg CRND8 mouse model (Chishti et al., 2001) (a gift from David Westaway, University of Alberta, Canada) with primers that converted the original Indiana mutation V717F into the Iberian mutation I716F (point mutation numbering is for the standard human APP 770 amino acid isoform). The N-terminus of APP^Swe/Ibe^ 695 was amplified using the forward primer 5′-GGCCGGCCGCCACCATGCTGCCCGGTTTGGCACT-3′ and reverse primer 5′-AAGGTGATGACGAACACTGTCG-3′; the C-terminus was amplified using the forward primer 5′-CGACAGTGTTCGTCATCACCTT-3′ and reverse primer 5′-GCGCGACGCGTCTAGTTCTGCATCTGCTCAAAGAA-3′. The resulting hAPP^Swe/Ibe^ fragments were combined by amplification using the forward primer 5′-GGCCGGCCGCCACCATGCTGCCCGGTTTGGCACT-3′ and reverse primer 5′-GCGCGACGCGTCTAGTTCTGCATCTGCTCAAAGAA-3′. The complete hAPP^Swe/Ibe^ 695 cassette was digested with MluI and FseI, and cloned into an Ai3-derived ROSA26 targeting vector (Madisen et al., 2010) that had been cut with the same enzymes to generate the final transgene construct pJJ-hAPP^Swe/Ibe^. The modified Ai3 targeting vector retained the original Cre-dependent CAG-loxP-stop-loxP sequence but removed the PGK-DTA negative selection cassette and shortened the 3′ targeting arm to reduce the overall vector size. All restriction enzymes were purchased from New England Biolabs (Ipswich, MA, USA).

### Embryonic stem cell targeting, screening, blastocyst injection and founder identification

The pJJ-hAPP^Swe/Ibe^ clone was linearized with BsrG1, gel purified and electroporated with CRISPR-assisted targeting vectors into agouti JM8.N4 embryonic stem (ES) cells (C57BL/6N background). Targeted cells were selected with G418, and 48 clones were screened by PCR for correct insertion of the ROSA26 construct. We confirmed 5′ targeting with the forward primer located within the ROSA26 5′-targeting arm, 5′-AGAAGAGGCTGTGCTTTGG-3′, and the reverse primer within the CAG promoter, 5′-TGGCGTTACTATGGGAACATAC-3′, yielding a 1340 bp band if correctly targeted. Correct 3′ targeting was confirmed with the forward primer located within the PGK polyA sequence, 5′-CAGCCTCTGTTCCACATACA-3′, and the reverse primer in the ROSA26 3′ targeting arm, 5′-GTCAAGCCAGTCCAAGAGAA-3′. Three clones were expanded and injected into C57BL/6 albino blastocysts yielding 30 offspring. 15 chimeric animals were screened by PCR using the forward primer located within the APP cDNA, 5′-CCTTGGTGATGCTGAAGAAGA-3′, and the reverse primer located in the WPRE cassette, 5′-AAGCCATACGGGAAGCAATAG-3′; positive offspring yielded a 400 bp band. One positive male was used to establish the R26-APP^Swe/Ibe^ line that was maintained by backcross to C57BL/6J. The same PCR reaction was used to genotype offspring for presence of the transgene.

### Mice

*R26-APP^Swe/Ibe^* mice were created as described above. Homozygote R26-APP animals were generated by mating R26-APP with Nestin-Cre or Nestin-CreER driver lines, and then intercrossing the offspring (Cre or CreER;R26-APP x R26-APP). Animals were genotyped by PCR for homozygosity using the ROSA26 forward primer, 5′-GTCGCTCTGAGTTGTTATCAGT-3′; ROSA26 reverse primer, 5′-CACACACCAGGTTAGCCTTTA-3′; and ROSA26-APP reverse primer, 5′-GACGTCAATGGAAAGTCCCTAT-3′; to check for the wild-type allele at 251 bp or the transgenic allele at 374 bp. The R26-APP mouse line will be available from The Jackson Laboratory as JAX#037319.

*Nestin-Cre* mice were derived from The Jackson Laboratory strain #3771 (Tronche et al., 1999).

*CaMKCreER^T2^* mice (referred to here as CaMKIIα-CreER) were the kind gift of Richard Huganir, Johns Hopkins Medical Institute, Baltimore, MD, USA, from the lines described by Erdmann et al. (2007). We did not determine whether the line we received contained one, two or four copies of the CreER transgene described in the original publication.

*Thy1-CreER^T2^* mice (also known as SLICK-H) were purchased from The Jackson Laboratory, strain #12708 (Young et al., 2008), on a hybrid CD1;B6 background. This line was backcrossed to C57BL/6J for several generations before intercrossing to other lines.

*tetO-APP^swe/ind^* line 102 mice were described by Jankowsky et al. (2005) and are available as MMRRC stock #34845-JAX.

*ztTA* mice (also known as ROSA26-ZtTA) were the kind gift of Liqun Luo and David C.-H. Wang, Stanford University and are available as Jax strain #12266 (Li et al., 2010).

*ROSA26:LNL:tTA* mice were purchased from The Jackson Laboratory, stock #11008 (Wang et al., 2008).

*Ai3* mice were purchased from The Jackson Laboratory strain #7903 (Madisen et al., 2010).

*Ai14* mice were purchased from The Jackson Laboratory strain #7914 (Madisen et al., 2010).

*CaMK2α-tTA* mice (referred to here by their prior name, CaMKIIα-tTA) were derived from The Jackson Laboratory strain #003010 (Mayford et al., 1996).

*5×FAD* mice were purchased from The Jackson Laboratory, stock #34848 (Oakley et al., 2006).

All lines except ztTA were maintained by backcrossing on a C57BL/6J background. ztTA was maintained on an ICR outbred background. Offspring for analysis were used either on a pure C57BL/6J background (all Cre/CreER;R26-APP animals), on a mixed ICRB6 background (all CreER;tTA;tetO-APP animals) or on a hybrid FVBB6 F1 background (CaMKIIα-tTA;tetO-APP animals). Animals of both sexes were used for all experiments; no animals were excluded from analysis.

All animal work was reviewed and approved by the Baylor College of Medicine Institutional Animal Care and Use Committee.

### Tamoxifen administration

Tamoxifen (Sigma, #T5648-5G) was administered intraperitoneally at a dose of 80-180 mg/kg once per day for five consecutive days. CaMKIIα-CreER;Ai3 mice in Fig. S1 were treated with 80 mg/kg, and Thy1-CreER;Ai14 mice in Fig S2 were treated with 120 mg/kg or 180 mg/kg; all other animals were treated with 180 mg/kg. Tamoxifen solution was made fresh each day. Triple transgenic CreER;tTA;APP mice were treated at 8 weeks of age and were harvested 14 days after the first tamoxifen injection. Young CaMKIIα-CreER;R26-APP mice were treated at 7-10 weeks of age and harvested 7-25 days after their final tamoxifen injection for western blotting, or at 9 and 18 months after injection for histology. Aged CaMKIIα-CreER;R26-APP mice were treated at ages ranging from 8-14 months and were harvested 7-14 days after their final tamoxifen injection.

### Tissue harvest and sectioning

R26-APP mice to be used for histology were killed by pentobarbital overdose and transcardially perfused with phosphate buffered saline (PBS) followed by 4% paraformaldehyde (PFA) in PBS. The brain was removed and post-fixed by immersion in PBS containing 4% PFA at 4°C overnight, and then cryoprotected by immersion in 30% sucrose in PBS at 4°C until equilibrated. R26-APP and tetO-APP mice to be used for biochemistry were killed by CO_2_ asphyxiation and dissected to isolate the cortex and hippocampus, which were snap frozen on dry ice and stored at −80°C until use. Fixed tissue was frozen on dry ice and sagittally sectioned at 35 µm thickness using a freezing-sliding microtome. Sections were stored in cryoprotectant media at −20°C until use.

### Western blotting

Samples were homogenized by sonication in either five volumes of PBS or 10 volumes of RIPA buffer (2.5-5 mM EDTA, 0.5% NP-40, 0.5% deoxycholate, 0.2% SDS in 1× PBS) with protease inhibitor (Roche, #5892970001). Samples homogenized in PBS were mixed 1:1 in 2× RIPA buffer containing protease inhibitor (1× PBS, 1% NP-40, 1% deoxycholate, 2% SDS, 5 mM EDTA) to put samples in a final buffer of 1× RIPA. Samples were spun for 10 min at 16,000 ***g*** and 4°C, and the supernatant was stored at −80°C until use. Approximately 30 μg of protein was diluted with 6× Laemmli buffer, denatured at 95°C for 5 min and electrophoresed on 4-15% Criterion TGX gels (Bio-Rad, #5671085). Proteins were transferred to nitrocellulose using the Trans-Blot Turbo Transfer System (Bio-Rad, #1704271). Membranes were blocked in TBS containing 5% non-fat dry milk for 1 h at room temperature (RT) and probed overnight at 4°C in blocking solution containing mouse anti-human β-amyloid antibody 6E10 (1:3000, BioLegend, #803001), rabbit anti-APP antibody Y188 (1:5000, Abcam, #ab32136) and chicken anti-GAPDH antibody (1:5000, Millipore, #AB2302). Antibodies were validated by confirming expected molecular weight on blots for GAPDH, or by past publication for APP (Baghallab et al., 2018; Guo et al., 2012; Pirttila et al., 1994). Membranes were washed with 1× TBS containing 0.1% Tween-20 and incubated for 1 h at RT in blocking solution containing secondary antibodies 680RD donkey anti-mouse IgG (Li-Cor #926-68070), 800RD donkey anti-rabbit IgG (Li-Cor #926-3221) and 680RD donkey anti-chicken IgG (Li-Cor, #926-68075) diluted 1:10,000. Membranes were again washed in TBS containing 0.1% Tween-20, briefly washed in TBS, then imaged on an Odyssey FC (Li-Cor) or a ChemiDoc MP imaging system (Bio-Rad). Proteins were quantified using Image Studio Lite (Li-Cor) or Image Lab (Bio-Rad) and normalized to GAPDH.

### Amyloid immunostaining

Brain sections were washed in 1× TBS to remove cryoprotectant before being blocked for 1 h at RT in 1× TBS containing 0.3% Triton X-100, 1% non-fat dry milk and 10% normal goat serum. Anti-human β-amyloid antibody 6E10 was diluted in blocking solution without milk overnight at 4°C (1:1000, BioLegend, #803001). The following day, sections were washed in 1× TBS and then incubated in secondary antibody diluted in blocking solution without milk for 2 h at RT (Alexa Fluor 488 goat anti-mouse IgG1, 1:500, Invitrogen, #A-21121). Sections were washed in 1× TBS, mounted and coverslipped using Prolong Diamond antifade media (ThermoFisher, #P36970).

### Amyloid quantification

Images were acquired using a Zeiss Axio Scan.Z1 at 5× magnification (Carl Zeiss AG, Oberkochen, Germany). Sections were imaged at a constant exposure time and lamp intensity. Two sagittal sections near 0.96 mm and 1.68 mm from bregma were chosen for quantification (Franklin and Paxinos, 2008). The cortex was outlined for analysis using ImageJ version 6. Images were converted from 16- to 8-bit and a fixed threshold was applied under the default IsoData algorithm to determine the percent area above threshold. Values from the two sagittal sections were averaged for each brain.

### Immunofluorescence and Thioflavin S staining

Brain tissue was washed in 1× TBS and blocked for 1.5 h at RT in 1× TBS containing 0.3% Triton X-100 and 5% normal goat serum. Brain sections were incubated overnight at 4°C in anti-GFAP (1:1000, Agilent/Dako, #Z033429-2) or anti-Iba1 primary antibody (1:1000, Wako, #019-1974) diluted in blocking solution. The next day, brain sections were washed in 1× TBS, incubated for 2 h at RT in secondary antibody diluted in blocking solution (1:500, Alexa Fluor 594 goat anti-rabbit, Invitrogen, #A-11037) and washed again in 1× TBS. Sections were then incubated in 0.002% Thioflavin S (Sigma, #T1892) in 1× TBS for 8 min, washed twice in 50% ethanol for 1 min and washed once more in 1× TBS for 5 min. Sections were mounted and coverslipped using Prolong Diamond antifade media (ThermoFisher, #P36970). Images were acquired using a Zeiss Axio Scan.Z1 at 20× magnification (Carl Zeiss AG, Oberkochen, Germany).

### Graph preparation

Graphs were prepared using GraphPad Prism 8. All graphs show mean±s.e.m. No statistical methods were used.

## Supplementary Material

Supplementary information
