# Supplementary material for: Temporal and spatially controlled APP transgene expression using Cre-dependent alleles
Source: Dis Model Mech. 2022 May 13;15(5):dmm049330. doi: 10.1242/dmm.049330 (PMC9118045; doi:10.1242/dmm.049330)
Supplement: Supplementary information [file dmm-15-049330-s1.pdf]

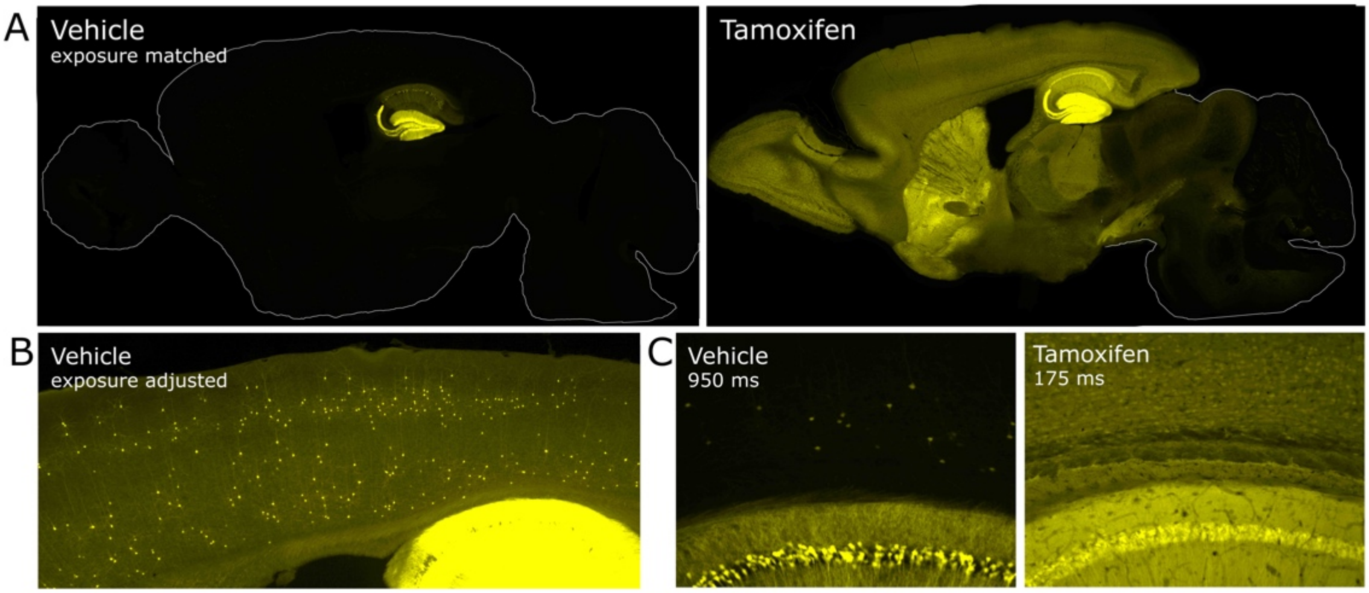

Fig. S1. Characterizing transgene induction in the CaMKIIα-CreER line using the Ai3 eYFP Cre reporter. Bigenic mice were treated with tam for 5 d at a dose of 80 mg/kg i.p. starting at approximately 3.5 mo. Animals were harvested 10-16 d after the first dose. **A**. Bigenic CaMKIIα-CreER;Ai3 animals treated with vehicle (left) have a noticeable degree of tam-independent reporter expression in the hippocampus, particularly in the dentate gyrus. This reporter line is sensitive and brightly fluorescent; different Cre-responder lines may show less leak in the absence of tam. Tam treatment induced strong fluorescence throughout the forebrain but none in the cerebellum or hindbrain, consistent with the expected pattern of CaMKIIα expression (right). Images shown in these panels were taken at identical exposure times. **B**. Higher magnification image showing cortex overlying the hippocampus from the vehicle-treated animal shown in panel A, but with the exposure increased to illustrate the smattering of cortical neurons that expressed eYFP in the absence of tam. **C.** Comparison of expression levels in CA1 and overlying cortex in vehicle- vs. tam-treated animals. The vehicle treated section required >4x the exposure time to match the fluorescence intensity of the tam-treated tissue.

Disease Models & Mechanisms • Supplementary information

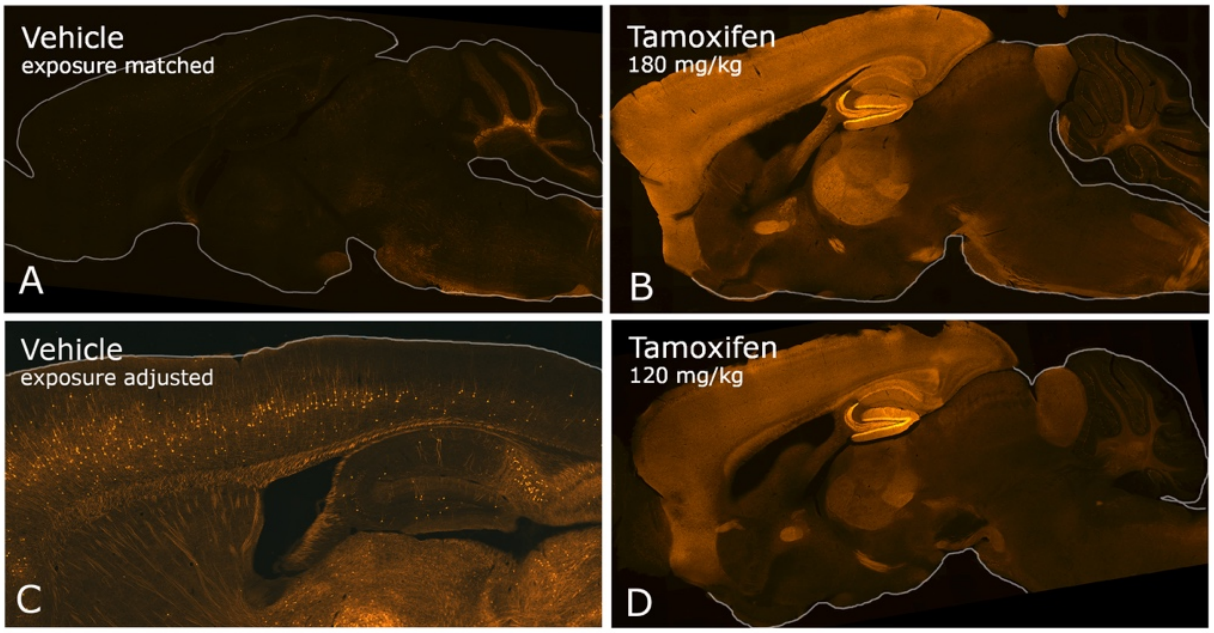

Fig. S2. Characterizing transgene induction in the Thy1-CreER line using the Ai14 tdTomato Cre reporter. Young adult bigenic mice were treated with tam for 5 d at 120 or 180 mg/kg i.p. Animals were harvested 1-2 weeks after the first dose. **A**. Bigenic Thy1-CreER;Ai14 animals treated with vehicle show limited reporter expression in the absence of tam. **B**. Tam treatment induced strong fluorescence throughout the forebrain and thalamus with additional expression noted in Purkinje neurons. Images shown in A and B were taken at identical exposure times. **C.** Higher magnification image of a separate vehicle-treated animal, but with the exposure increased to illustrate the smattering of cortical neurons that expressed tdTomato in the absence of tam. The Thy1-CreER line showed roughly the same level of cortical leak as the CaMKIIα-CreER, but considerably less tam-independent expression in the hippocampus. **D**. Treatment at 120 mg/kg for 5 d induced the same pattern of reporter expression as 180 mg/kg. Doses as low as 90 mg/kg were also effective.

Disease Models & Mechanisms • Supplementary information

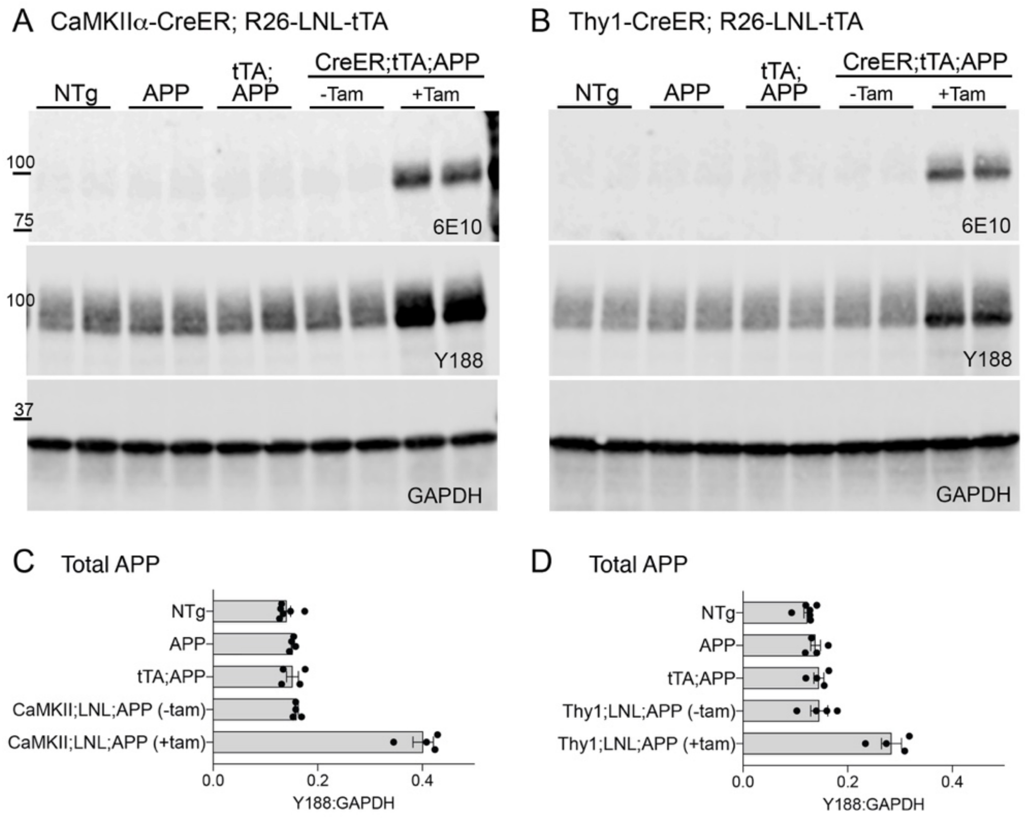

Fig. S3. Hippocampal APP expression parallels cortical levels in triple transgenic Cre-dependent APP models. A-B Western blots for human APP (6E10), total APP (Y188), and internal control GAPDH on hippocampal homogenates from each of the ROSA-LNL-tTA combinations tested: CaMKIIα-CreER; ROSA-LNL-tTA;tetO-APP (**A**) and Thy1-CreER;ROSA-LNL-tTA;tetO-APP (**B**). Each blot includes 4 genotypes: NTg, tetO-APP single Tg (APP), ROSA-LNL-tTA + tetO-APP double Tg (tTA;APP), and the triple transgenic CreER + ROSA-LNL-tTA + tetO-APP (CreER;tTA;APP). Triple transgenic mice were harvested with and without tamoxifen treatment of 180 mg/kg for 5d (+/- tam). **C-D**. Quantitation of total APP (Y188) relative to GAPDH for each model: CaMKIIα-CreER;ROSA-LNL-tTA;tetO-APP (**C**) and Thy1-CreER;ROSA-LNL-tTA;tetO-APP (**D**). n=4 mice for all groups except NTg n=6. Graphs show mean ± s.e.m.

Disease Models & Mechanisms • Supplementary information

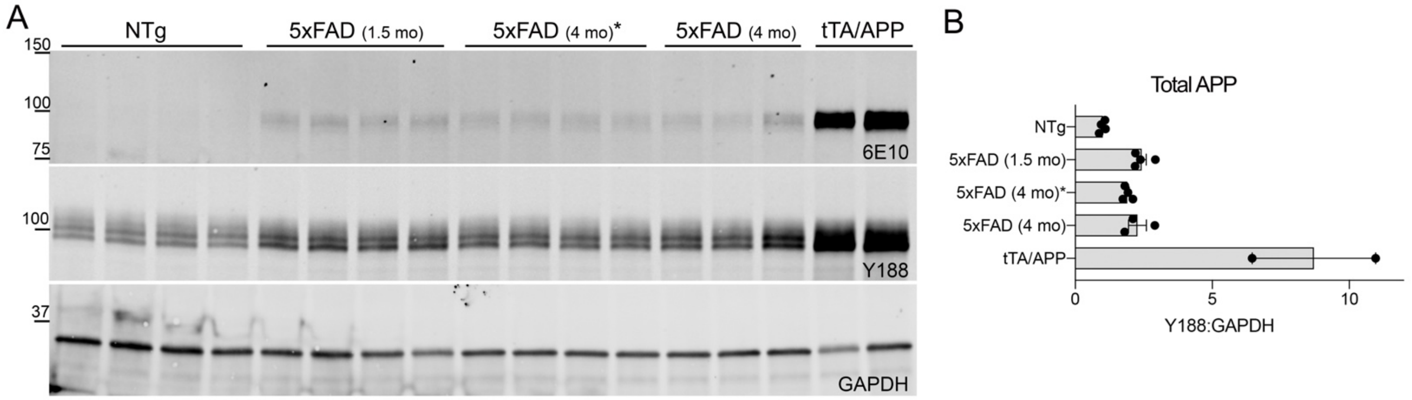

Fig. S4. APP expression in the 5xFAD line appears lower now than originally described in two independent colonies. A. Protein expression in cortical homogenates was measured by Western blot for human APP (6E10), total APP (Y188), and GAPDH from 5xFAD and CaMKIIα-tTA;tetO-APP mice (tTA;APP). Tissue was harvested at 2 time points to determine if expression increased with age, and from two independent mouse colonies to determine if copy loss had occurred in our original cohort. Left to right: NTg, 5xFAD (1.5 mo), 5xFAD (4 mo)* from a second colony, 5xFAD (4 mo), and CaMKIIα-tTA;tetO-APP mice. **B**. Quantitation of total APP relative to GAPDH, normalized to NTg. n=2-4 mice per genotype. Graph shows mean ± s.e.m.

Disease Models & Mechanisms • Supplementary information
